# Prevalence of malaria parasitaemia among pregnant women at booking in Nigeria

**DOI:** 10.1002/hsr2.1337

**Published:** 2023-06-09

**Authors:** Olufiade P. Oyerogba, Aduragbenro Adedapo, Titilayo Awokson, Akin‐Tunde Odukogbe, Nicholas Aderinto

**Affiliations:** ^1^ Department of Obstetrics and Gynaecology University College Hospital Ibadan Nigeria; ^2^ Department of Medicine and Surgery Ladoke Akintola University of Technology Ogbomoso Nigeria

**Keywords:** Africa, malaria, Nigeria, pregnancy, prevalence, Sub‐Saharan Africa

## Abstract

**Background:**

Malaria is a major public health concern among pregnant women in sub‐Saharan Africa. Within the region, Nigeria has the highest malaria cases. This study aimed to determine the prevalence and factors associated with malaria parasitaemia among pregnant women at a booking clinic in Ibadan, Nigeria.

**Methods:**

A cross‐sectional study was conducted between January and April 2021 at the University College Hospital in Ibadan, Nigeria. A sample of 300 pregnant women participated, and anaemia and malaria were diagnosed using packed cell volume and Giemsa‐stained blood smears, respectively. Data analysis was done using SPSS 25.0.

**Results:**

The study found that 26 (8.70%) pregnant women tested positive for malaria parasitaemia. Factors such as age, religion, level of education, and occupation were significantly related to the prevalence of malaria parasitaemia among pregnant women with *p* < 0.05.

**Conclusion:**

Our study identified a high prevalence of malaria parasitaemia among pregnant women with demographic factors such as age, religion, level of education, and occupation significantly associated. Targeted malaria control interventions for pregnant women with low levels of education and low‐income occupations are necessary, with more research needed to evaluate their effectiveness.

## 1 INTRODUCTION

Malaria poses a severe health threat in sub‐Saharan Africa, particularly for pregnant women. The World Health Organization reports that in 2018, approximately 228 million new malaria cases were reported worldwide, with most of them in Africa.^1^ Nigeria was responsible for nearly 24% of all global malaria‐related deaths in 2018.^1^

Pregnancy increases the risk of severe disease for women infected with malaria due to immune system suppression.^2^ This puts them at a three times higher risk of severe illness and a death rate close to 50%.^2^ The risk varies with the woman's age and the number of pregnancies. In areas with high malaria transmission rates, 25% of pregnant women are infected.^3^ Malaria during pregnancy can result in serious consequences for both the mother and the fetus, including intrauterine growth restriction, congenital malaria, low birth weight, abortion, and stillbirth. Symptoms and complications vary according to the level of immunity and the intensity of transmission in the area.

Most infections in pregnancy are asymptomatic, undetected, and untreated, leading to negative outcomes for both the mother and the foetus.^4^ Women in their first and second pregnancies are the most vulnerable to Plasmodium falciparum infection.^5^ This is due to increased immunity with more pregnancies. Severe malaria can lead to maternal death from complications such as hypoglycemia, cerebral malaria, and pulmonary oedema.^6^ The burden of malaria during pregnancy also causes unwanted consequences, prolonged hospital stays, and psychological trauma for both patients and healthcare providers.^7^

Pregnant women in Nigeria are particularly vulnerable to malaria infection due to various factors such as poor access to quality healthcare services, poverty, and demographic characteristics.^8^ The burden of malaria is highest in rural areas, where access to health services is limited. The poverty rates in these areas make it challenging for pregnant women to access healthcare services and afford preventive measures such as insecticide‐treated bed nets and antimalarial drugs.^9^ This lack of access to quality healthcare services also means that many cases of malaria go undiagnosed and untreated, leading to severe complications for pregnant women and their fetuses.

Studies have found that demographic factors such as age, level of education, and occupation are significantly associated with malaria prevalence among pregnant women in Nigeria.^10^, ^11^ Younger pregnant women, those with low levels of education, and low‐income occupations are more likely to be affected by malaria than their older, more educated, and higher‐income counterparts.^12^ These findings emphasise the need for targeted interventions that consider these demographic factors to effectively control the burden of malaria among pregnant women in Nigeria. Despite efforts to reduce the burden of malaria in Nigeria, the disease remains a significant challenge, particularly among vulnerable populations such as pregnant women. Continued investment in malaria prevention and control measures, including targeted interventions for pregnant women, is crucial to ensure the health and well‐being of this vulnerable population.

The current prevalence of malaria in pregnancy at the University College Hospital in Ibadan booking clinic is unknown. A prospective review of malaria in pregnancy protocols is necessary to provide updated evidence and information. The results of this study will contribute to developing an evidence‐based protocol for malaria during pregnancy in this clinical setting and similar settings in Nigeria, sub‐Saharan Africa and beyond.

## 2 RESEARCH METHODOLOGY

### 2.1 Study area

The study was carried out at the Department of Obstetrics and Gynaecology Department of University College Hospital (UCH) in Ibadan, Nigeria, during the first quarter of 2021. UCH is a tertiary care medical center linked with the College of Medicine at the University of Ibadan, which was established in 1952 and had 850 available beds. The Obstetrics and Gynaecology department, one of the largest in the hospital, has 24 consultants and 29 resident doctors in training, with 180 total beds, 120 for obstetrics and 60 for gynaecology. The department typically sees 2000–2400 annual prenatal appointments and delivers 2500–3000 births, with 900–1100 spontaneous vaginal deliveries and an average of 1250–1500 C‐sections, with 750–850 as emergency and 500–600 as elective procedures.

### 2.2 Study design

This study was designed as a cross‐sectional investigation conducted at the antenatal clinics of University College Hospital in Ibadan, Nigeria. The study aimed to determine the prevalence of malaria parasitaemia among pregnant women at booking and identify demographic factors associated with the prevalence of the disease. Ethical clearance was obtained from the Ethics Committee of the University of Ibadan/University College Hospital before the commencement of the study.

A simple random sampling method was used to recruit participants for the study. The process involved assigning a unique identifier to each pregnant woman attending the antenatal clinic at the University College Hospital in Ibadan. The identifiers were then entered into a randomization tool, a Microsoft Excel sheet, which generated a list of 300 identifiers. The list of identifiers was used to recruit participants from the antenatal clinic until the desired sample size of 300 was achieved. This method ensured that each pregnant woman had an equal chance of being selected for the study without any biases or pre‐selection based on any characteristics. Using a simple random sampling method, the study aimed to ensure that the sample was representative of the pregnant women attending the antenatal clinic at the University College Hospital in Ibadan.

Women were eligible for participation if they attended the antenatal clinic and agreed to participate. However, those who had already received antimalarial treatment or prevention, those who declined to participate, or those with sickle cell anaemia were excluded from the study. The principal investigator verified each participant's eligibility and obtained their informed consent, and the allocation numbers were recorded on the participant's forms for data analysis purposes.

Informed consent was obtained from each participant before the beginning of data collection. The process of obtaining informed consent was conducted thoroughly, which involved explaining the study's purpose and procedures, including the risks and benefits of participation. Participants were informed of their right to refuse or withdraw from the study at any time without consequence. During the informed consent process, potential participants received a copy of the informed consent form to read and sign. For participants who could not read or write, the informed consent form was read aloud and explained in detail by the research team. Participants who agreed to participate in the study were asked to sign or thumbprint the informed consent form.

Data collection was carried out using a structured questionnaire and laboratory investigations. The questionnaire was designed to collect detailed demographic characteristics such as age, religion, level of education, and occupation. The laboratory investigations utilized the gold‐standard microscopy method to test for malaria parasitaemia, the recognized diagnostic technique for detecting malaria.

The study considered two main hypotheses:

1. The prevalence of malaria parasitemia among pregnant women at booking in Nigeria is high at the time of booking.
2. Sociodemographic factors such as age, religion, level of education, and occupation are associated with malaria parasitemia among pregnant women at booking in Nigeria.

### 2.3 Study plan

The study obtained blood samples from pregnant women visiting the antenatal booking clinics who met eligibility criteria and provided informed consent. The samples were collected by the head researcher and sent to a laboratory for examination. The principal investigator then refined and analysed the information using participant data forms.

### 2.4 Sample size determination

The sample size of 300 was calculated using the Cochrane formula^13^

$$N=\frac{Z^{2}\times p\left(1-p\right)}{d^{2}},$$

where

*N* = minimum sample size

Z = standard score corresponding to a given confidence level. At a 95% confidence level or 5% significance level (0.05), Z = 1.96.

p = prevalence of malaria parasitemia and prevalence of 38.8% in a previous study in Northcentral Nigeria was used^14^

d = precision limit or proportion of sampling error at 6% (0.06)

*N* = 275.392

*N* = 275

Considering an attrition rate of 10% = 25.3

Total sample size = 275 + 25 = 300.

### 2.5 Data analysis

Statistical analysis was conducted using the Social Sciences Statistical Package (SPSS‐25.0). The resulting data was analysed to generate tables, percentages, and Chi‐square tests.

### 2.6 Ethical considerations

The study was granted ethical approval from the Institutional Review Board of the University of Ibadan/University College Hospital. Participants were thoroughly informed about the study procedures and allowed to participate voluntarily. They were assured that declining to participate would not impact their future medical care. The study team obtained written consent from all individuals who agreed to participate.

## 3 RESULTS

This study investigated the prevalence of malaria parasitaemia among pregnant women using a sample of 300 patients drawn at the University College Hospital, Ibadan booking clinic. Table 1 shows the demographic characteristics of the patients enrolled in the study. The largest proportion of participants, 102 (33.7%), fell within the age range of 31–35 years. A significant majority, 289 (96.3%) of the respondents, were married, while the sample comprised a greater proportion of Christians, 216 (72.0%), compared to Muslims, 84 (28.0%). The majority of the participants, 248 (82.7%), were of Yoruba ethnicity, which could be attributed to the study's location. In particular, patients exhibited a high level of education, with 278 (92.7%) holding a tertiary institution certificate. Additionally, 96 (32.0%) of the sample reported being members of a professional organization, indicating a highly professional population.

**Table 1:** Socio‐demographic and economic characteristics of the respondents.

Variables	Categories	Frequency	Percent
Age (years)	<25	26	8.7
	26–30	77	25.7
	31–35	102	33.7
	>35	95	34.0
Marital status	Married	289	96.3
	Single	11	3.7
Religion	Christianity	216	72.0
	Islamic	84	28.0
Tribe	Yoruba	248	82.7
	Hausa	1	0.3
	Igbo	26	8.7
	Others	25	8.3
Level of education	None	1	0.3
	Primary	1	0.3
	Secondary	20	6.7
	Tertiary	278	92.7
Occupation	Civil servant	50	16.7
	Trader	23	7.7
	Business	49	16.3
	Professional	96	32.0
	Housewife	2	0.7
	Others	80	26.7

Table 2 describes the obstetric history of the patients under investigation. The table reveals that 123 (41.0%) of the participants were nullipara, 85 (28.3%) were primipara, and the remaining 92 (30.7%) were multipara. In particular, all the respondents were booked patients since they were recruited from the antenatal clinic. Additionally, the data reveal that 134 (44.7%) of the participants booked their pregnancy during the second trimester, 116 (38.7%) during the third trimester, and the remaining 50 (16.7%) during the first trimester. These findings suggest that most patients book their pregnancy during the second trimester. Regarding the date of the last pregnancy, the majority of respondents, 136 (45.3%), reported having had their previous child within the last 2 years, while 123 (41%.0) were experiencing their first pregnancy. These results indicate that the study population included experienced and inexperienced pregnant women.

**Table 2:** Obstetrics history of the respondents.

Variables	Categories	Frequency	Percent
Parity	Nullipara	123	41.0
	Primipara	85	28.3
	Multipara	92	30.7
Booking status	Un‐booked	82	27.3
Gestational age at booking	1st Trimester	50	16.7
	2nd Trimester	134	44.7
	3rd Trimester	116	38.7
Last pregnancy (years)	0	123	41.0
	1–2	136	45.3
	3–4	37	12.3
	>4	4	1.3

Table 3 shows the findings of the secondary outcome, namely the risk of malaria exposure among the respondents. The results indicate that the vast majority, 266 (88.7%) of the participants, were not anaemic, suggesting they have a low risk of malaria exposure. Furthermore, the data indicates that most of the patients, 181 (60.3%), did not sleep under insecticide‐treated nets. Additionally, most of the respondents, 177 (59.0%), did not take antimalaria medication (Fansider) during their pregnancy. Interestingly, only a small proportion, 51 (17.0%), reported experiencing malaria symptoms during their pregnancy. Lastly, the malaria test conducted during the study revealed that only 26 (8.7.0%) of the patients tested positive for the infection. Furthermore, among the 26 patients who tested positive for malaria, 11 (42.3%) were primigravida, 4 (15.4%) were primipara, and the remaining 11 (42.3%) were multigravida. These findings suggest that primigravida and multigravida may have a higher risk of malaria than primipara in the sample population. Further details can be found in Table 3. Similarly, of the 34 patients who were anaemic, only 6 (17.7%) tested positive for malaria, indicating that nonmalaria‐related anaemia is more prevalent among pregnant women at the booking stage.

**Table 3:** Secondary outcome: risk of exposure to malaria of the respondents.

Variables	Categories	Frequency	Percent
PCV	Anaemic	34	11.3
	Nonanaemic	266	88.7
Sleeping with net	Yes	119	39.7
	No	181	60.3
Took fansider drug	Yes	123	41.0
	No	177	59.0
Had history of malaria symptoms?	Yes	51	17.0
	No	249	83.0
Test for malaria	Positive	26	8.7
	Negative	274	91.3
Malaria positive patients	Primigravida	11	3.7
	Primipara	4	1.3
	Multigravida	11	3.7

The social‐demographic characteristics of the respondents significantly associated with the prevalence of malaria parasitaemia among pregnant women at the booking stage are presented in Table 4. According to the Chi‐square results, age, religion, level of education, and occupation were found to be statistically related to the prevalence of malaria parasitaemia among pregnant women (*χ* ^2^ = 299.000, *p* < 0.05; *χ* ^2^ = 10.942, *p* < 0.05; *χ* ^2^ = 249.347, *p* < 0.05; and *χ* ^2^ = 141.811, *p* < 0.05). In contrast, factors such as marital status and tribe were not significantly related to the prevalence of malaria parasitemia.

**Table 4:** Relationship between the sociodemographic characteristics and the prevalence of malaria parasitaemia among pregnant women.

Variable	Categories	Prevalence of malaria parasitaemia among pregnant women		*χ* ^2^	*df*	*p* value
		Nonprevalent	Prevalent			
Age (years)	<25	0	26 (100.0%)	299.000	3	**0.000** a
	26–30	77 (28.2%)	0			
	31–35	102 (37.4%)	0			
	>35	94 (34.4%)	0			
Marital status	Married	263 (96.3%)	26 (100.0%)	.985	1	0.321
	Single	10 (3.7%)	0			
Religion	Christianity	190 (69.6%)	26 (100.0%)	10.942	1	**0.001** a
	Islamic	83 (30.4%)	0			
Tribe	Yoruba	222 (81.3%)	26 (100.0%)	5.856	3	0.119
	Hausa	1 (0.4%)	0			
	Igbo	26 (9.5%)	0			
	Others	24 (8.8%)	0			
Level of education	None	0	1 (3.8%)	249.347	3	**0.000** a
	Primary	0	1 (3.8%)			
	Secondary	0	20 (76.9%)			
	Tertiary	273 (100.0%)	4 (15.4%)			
Occupation	Civil servant	24 (8.8%)	26 (100.0%)	141.811	5	**0.000** a
	Trader	23 (8.4%)	0			
	Business	49 (17.9%)	0			
	Professional	96 (35.2%)	0			
	Housewife	2 (0.7%)	0			
	Others	79 (28.9%)	0			

*Note*: *χ* ^2^ = Pearson Chi square value, *df* = degree of freedom, *p* = probability value.

a Significant at *p* < 0.050.

Table 5 presents the results of the relationship between obstetric history and the prevalence of malaria parasitaemia among pregnant women at the booking stage. The study found a statistically significant relationship between parity, gestational age at booking, and the date of last pregnancy with malaria parasitaemia, with Chi‐square values of (*χ* ^2^ = 40.746, *p* < 0.05; *χ* ^2^ = 141.811, *p* < 0.05; and *χ* ^2^ = 40.746, *p* < 0.05). Nullipara and multipara were also observed to have a higher number of nonprevalent malaria parasitaemia compared to primipara.

**Table 5:** Relationship between the obstetrics history and the prevalence of malaria parasitaemia among pregnant women.

Variables	Categories	Prevalence of malaria parasitaemia among pregnant women		*χ* ^2^	*df*	*p* value
		Nonprevalent	Prevalent			
Parity	Nullipara	97 (35.5%)	26 (100.0%)	40.746	2	**0.000** a
	Primipara	85 (31.1%)	0			
	Multipara	91 (33.3%)	0			
Booking status	Un‐booked	56 (100.0%)	26 (100.0%)	–	–	–
Gestational age at booking	1st Trimester	24 (8.8%)	26 (100.0%)	141.811	2	**0.000** a
	2nd Trimester	134 (49.1%)	0			
	3rd Trimester	115 (42.1%)	0			
Last pregnancy (years)	0	97 (35.5%)	26 (100.0%)	40.746	3	**0.000** a
	1–2	136 (49.8%)	0			
	3–4	37 (13.6%)	0			
	>4	3 (1.1%)	0			

*Note*: *χ* ^2^ = Pearson Chi square value, *df* = degree of freedom, *p* = probability value.

a Significant at *p* < 0.050.

## 4 DISCUSSION

The study aimed to assess the prevalence of malaria parasitaemia among pregnant women during their first prenatal visit to University College Hospital in Ibadan. The study considered the sociodemographic characteristics and pregnancy history. The findings revealed that 8.7% of pregnant women had malaria parasitaemia. This figure is comparable to the rate found in Ethiopia but lower than that in Ghana, Burkina Faso, and Malawi, which reported rates of 10.2%, 20.4%, 18.1%, and 19%, respectively.^15^, ^16^, ^17^ In contrast, other studies conducted in Nigeria showed different rates, with Lagos reporting 7.7% and Katsina recording 53.9%.^10^, ^18^ These findings significantly affect malaria control programmes, especially those focused on pregnant women. Based on the local epidemiological situation, the study highlights the need for targeted interventions to address malaria in pregnant women. Targeted interventions can lead to better health outcomes for pregnant women and their newborns, reducing the burden of malaria in affected communities and contributing to achieving global health targets such as the Sustainable Development Goals.

Our study investigates the social demographic factors that may have a significant relationship with malaria parasitemia in pregnant women. Our findings show that the patients’ education level and occupation were significantly associated with malaria parasitaemia. In particular, higher levels of education can lead to increased health awareness, which may contribute to a lower prevalence of malaria. This finding is consistent with previous research, where women with a high school education or higher were 0.53 times less likely to have malaria than those without education.^19^ Similarly, we found that the type of occupation had a significant correlation with the prevalence of malaria. Our study revealed that professionals such as doctors, lawyers, accountants, and engineers were less likely to have malaria parasitaemia. This finding is consistent with a study by Dosoo et al.,^5^ which reported that the least poor were 0.34 times less likely to have malaria parasitaemia than the poorest. This highlights the importance of social demographic factors in determining the prevalence of malaria parasitemia in pregnant women. It suggests that interventions aimed at improving health education and increasing the income levels of pregnant women may effectively reduce the burden of malaria in this population. Further studies are needed to investigate the mechanisms underlying these relationships and develop effective interventions.

This study investigated the relationship between parity and malaria prevalence among pregnant women. The results revealed a statistically significant association, with primigravida having a higher malaria prevalence than multigravida. This finding may be explained by the acquired immunity that develops with increasing parity, reducing susceptibility to malaria infection in multigravida. These findings are consistent with a study conducted in Uganda, where primigravid women had an 84% higher risk of microscopic parasitaemia at enrollment and nearly three times the risk of placental malaria compared to multigravida women.^20^ Additionally, Ugwu et al.^21^ reported a higher prevalence of malaria parasitaemia among primigravidae (95.8%) compared to multigravida (90.2%). The association between parity and malaria prevalence among pregnant women is important for malaria prevention and control strategies. Efforts to promote early antenatal care and ensure access to malaria prevention measures such as insecticide‐treated bed nets and chemoprophylaxis may be particularly important for primigravids. Future research is needed to understand better the mechanisms underlying the observed relationship between parity and malaria susceptibility among pregnant women.

The use of insecticide‐treated nets (ITNs) has been demonstrated to be a highly effective means of reducing the burden of malaria among pregnant women. However, in the current study, only 39.7% of participants reported using ITNs. This finding highlights the need to improve access to and utilization of ITNs in the study population. This result is consistent with a study by Njumkeng^22^ and colleagues in Cameroon, which reported a 32.7% and 57.4% reduction in the likelihood of malaria infection among irregular and regular ITN users, respectively, compared to nonusers. ITNs should be promoted as part of a comprehensive approach to malaria prevention and control among pregnant women. Furthermore, the current study aligns with the findings of Agyeman and colleagues, who reported a reduction in the prevalence of malaria parasitaemia due to self‐treatment of malaria in a study conducted in Northern Ghana.^23^ These findings underscore the importance of increasing health literacy and empowering pregnant women to take an active role in their health care.

## 5 LIMITATIONS OF STUDY

This study had some limitations. One potential limitation of this study is that it was conducted in a single hospital in Nigeria and, therefore, may not represent pregnant women in other regions or healthcare settings. Furthermore, the study relied on self‐reported participant data, which may introduce the possibility of recall bias or social desirability bias. Finally, the study did not include a comparison group of nonpregnant women or men, which limits the ability to conclude the prevalence of malaria parasitaemia in the general population. Statistical limitations of the study include the lack of adjustment for potential confounding factors in the analysis of the relationship between occupation and malaria parasitaemia and the lack of multivariable analysis to identify independent risk factors for malaria parasitaemia among pregnant women. Despite these limitations, the study's findings provide valuable information on the prevalence of malaria parasitaemia among pregnant women in Nigeria and its associated risk factors. These findings can guide the development of targeted interventions to reduce the burden of malaria in pregnancy and improve maternal and foetal outcomes.

## 6 CONCLUSION

The findings of our study demonstrate a noteworthy prevalence of malaria parasitaemia among pregnant women within the targeted study population, thus raising concerns regarding the health implications and management strategies associated with this condition. Demographic factors such as age, religion, level of education, and occupation were significantly associated with malaria parasitemia. Specifically, younger pregnant women, those with lower levels of education, and those in low‐income occupations were at a higher risk of malaria infection. To address this issue, targeted malaria control interventions are urgently needed for these high‐risk groups of pregnant women. These interventions can include increasing access to insecticide‐treated nets, providing regular malaria screening and treatment during prenatal visits, and promoting health education programmes focusing on preventing and managing malaria during pregnancy. However, further research is needed to evaluate these interventions’ effectiveness in reducing malaria burden among pregnant women. This could involve conducting randomized controlled trials to assess the impact of interventions on malaria prevalence and assessing the cost‐effectiveness of different interventions to inform policy decisions.

## AUTHOR CONTRIBUTIONS

**Olufiade P. Oyerogba**: conceptualization; data curation; formal analysis; writing—original draft; writing—review & editing. **Aduragbenro Adedapo**: conceptualization; formal analysis; project administration; writing—original draft; writing—review & editing. **Titilayo Awokson**: methodology; project administration; writing—original draft; writing—review & editing. **Akin‐Tunde Odukogbe**: conceptualization; methodology; project administration; supervision; writing—review & editing. **Nicholas Aderinto**: project administration; writing—review & editing.

## CONFLICT OF INTEREST STATEMENT

The authors declare no conflicts of interest.

## ETHICS STATEMENT

The study received ethical clearance from the University of Ibadan/University College Hospital Institutional Review Committee. Participants were informed of the study procedures and allowed to participate voluntarily. Those who chose not to participate would not be affected in their future care. Written consent was obtained from all participants before their inclusion in the study.

## TRANSPARENCY STATEMENT

The lead author Nicholas Aderinto affirms that this manuscript is an honest, accurate, and transparent account of the study being reported; that no important aspects of the study have been omitted; and that any discrepancies from the study as planned (and, if relevant, registered) have been explained.

## Data Availability

The data that support the findings of this study are available on request from the corresponding author. The data are not publicly available due to privacy or ethical restrictions. Data may be made available upon reasonable request from the corresponding author. The data are not publicly available due to privacy or ethical restrictions.
